# From Lesions to Viral Clones: Biological and Molecular Diversity amongst Autochthonous Brazilian *Vaccinia Virus*

**DOI:** 10.3390/v7031218

**Published:** 2015-03-16

**Authors:** Graziele Oliveira, Felipe Assis, Gabriel Almeida, Jonas Albarnaz, Maurício Lima, Ana Cláudia Andrade, Rafael Calixto, Cairo Oliveira, José Diomedes Neto, Giliane Trindade, Paulo César Ferreira, Erna Geessien Kroon, Jônatas Abrahão

**Affiliations:** 1 Laboratório de Vírus, Departamento de Microbiologia, Universidade Federal de Minas Gerais, Belo Horizonte, Minas Gerais 31270-901, Brazil; E-Mails: graziufmg@yahoo.com.br (G.O.); felipelopeasssis@gmail.com (F.A.); jonasalbarnaz@yahoo.com.br (J.A.); maurili15@hotmail.com (M.L.); ana.andrade2008@hotmail.com (A.C.A.); calixtomicro@yahoo.com.br (R.C.); gitrindade@yahoo.com.br (G.T.); peregrinopcp@hotmail.com (P.C.F.); ernagkroon@gmail.com (E.G.K.); 2 (AQUACEN) Laboratório Nacional Oficial de Referência de Doenças de Animais Aquáticos, Universidade Federal de Minas Gerais, Belo Horizonte, Minas Gerais 31270-901, Brazil; E-Mail: gabriel.magno@gmail.com; 3 Laboratório de Retroviroses, Departamento de Medicina Veterinária Preventiva, Escola de Veterinária da Universidade Federal de Minas Gerais, Belo Horizonte, Minas Gerais 31270-901, Brazil; E-Mail: cairo_henrique@yahoo.com.br; 4 Centro Agropecuário, Departamento de Ciência Animal, Universidade Federal do Pará, Pará 66075-110, Brasil; E-Mail: diomedes@ufpa.br

**Keywords:** *Vaccinia virus*, clones, diversity, evolution

## Abstract

*Vaccinia virus* (VACV) has had an important role for humanity because of its use during the smallpox eradication campaign. VACV is the etiologic agent of the bovine vaccinia (BV), an emerging zoonosis that has been associated with economic, social, veterinary and public health problems, mainly in Brazil and India. Despite the current and historical VACV importance, there is little information about its circulation, prevalence, origins and maintenance in the environment, natural reservoirs and diversity. Brazilian VACV (VACV-BR) are grouped into at least two groups based on genetic and biological diversity: group 1 (G1) and group 2 (G2). In this study, we went to the field and investigated VACV clonal diversity directly from exanthemous lesions, during BV outbreaks. Our results demonstrate that the G1 VACV-BR were more frequently isolated. Furthermore, we were able to co-detect the two variants (G1 and G2) in the same sample. Molecular and biological analysis corroborated previous reports and confirmed the co-circulation of two VACV-BR lineages. The detected G2 clones presented exclusive genetic and biological markers, distinct to reference isolates, including VACV-Western Reserve. Two clones presented a mosaic profile, with both G1 and G2 features based on the molecular analysis of A56R, A26L and C23L genes. Indeed, some SNPs and INDELs in A56R nucleotide sequences were observed among clones of the same virus population, maybe as a result of an increased mutation rate in a mixed population. These results provide information about the diversity profile in VACV populations, highlighting its importance to VACV evolution and maintenance in the environment.

## 1. Introduction

*Vaccinia virus* (VACV) has had an important role in human history due to its use as a vaccine during the smallpox vaccination campaign, resulting in the eradication of this deadly disease in 1980 [1]. More recently, VACV has been widely used as a vector for recombinant vaccines [2,3,4]. Following Smallpox eradication, other zoonotic orthopoxviruses (OPV) have emerged worldwide, such as *Cowpox virus* (CPXV), in Europe, *Monkeypox virus* (MPXV), endemic in many African countries and recently introduced in the USA, and finally VACV, endemic in Brazil and India [5,6,7,8,9,10,11,12,13]. VACV is the causative agent of bovine vaccinia (BV), an exanthemous disease responsible for outbreaks that affect both bovines and humans, causing public health impacts and economic losses in South America and India [10,11,12,14,15]. The clinical prognosis of BV includes the appearance of papules that progress to vesicles and growing scabs mainly on the teats and udder of infected bovines. In humans, the lesions occur primarily on milkers’ hands and arms. Other symptoms, such as fever, myalgia, headache, arthralgia and lymphadenopathy are also frequently reported [11,16]. During outbreaks, direct contact with infected animals is the main transmission route to humans, but it may also occur by contact with contaminated fomites. Furthermore, reports have suggested a possible human-to-human transmission of VACV [16,17,18].

In Brazil, VACV was first isolated from rodents in the 1960s and since 1999 BV outbreaks have been consistently reported [10,12,15,19,20,21,22,23,24]. Some reports have shown a great genetic and biologic heterogeneity among Brazilian VACV (VACV-BR) isolates. This variability allowed clustering of VACV-BR into at least two distinct groups (Group 1—G1 and Group 2—G2), demonstrating a dichotomy supported by evidence of molecular and biological diversity such as virulence in BALB/c mouse model and plaque phenotype in BSC-40 cells. Group 2 strains display higher plaque sizes and are virulent to mice, unlike Group 1 [24,25,26,27,28]. Moreover, polymorphisms observed in specific VACV genes, such as hemagglutinin gene (A56R), A-type inclusion body gene (A26L), and chemokine binding protein gene (C23L), have been used in phylogenetic studies and further confirmed the dichotomy between G1 and G2 VACV-BR [22,27,29,30].

The VACV-BR G2 comprises of viral isolates Guarani P1 virus (GP1V), Pelotas 1 virus (P1V), Belo Horizonte virus (VBH), the newest isolate Serro human virus 2 (SH2V) and others, while G1, the group most frequently reported during BV outbreaks, includes isolates of Araçatuba virus (ARAV), Cantagalo virus (CTGV), Mariana virus (MARV), Guarani P2 virus (GP2V), Pelotas 2 virus (P2V) and others [22,23,24,26,28,31,32]. It is worth mentioning that the GP1V and GP2V were isolated during the same outbreak (co-circulation), while P1V and P2V were isolated from the same clinical sample (co-infection) [26,28].

Several studies have demonstrated genetic and biological diversity among isolates of the same species of a given OPV, including *Variola virus* (VARV), CPXV and MPXV. Diversity studies among MPXV isolates have revealed the existence of a dichotomy supported by differences in epidemiological and clinical features, while CPXV is clustered in four or more groups. Furthermore, studies on VARV variability have demonstrated the existence of at least two groups with distinct virulence profiles: variola *major* and variola *minor*. Taken together, these studies showed a great biological and genetic diversity amongst isolates from different OPV species [33,34,35,36,37,38,39,40].

Osborne and colleagues in 2007 reported high genetic variability among clones of the smallpox vaccine Dryvax [41]. Moreover, other studies reveal that old vaccines were a complex mixture of viruses with a large number of single-nucleotide polymorphisms (SNPs) besides insertions and deletions (INDELs), resulting in genetically and phenotypically distinct clones [42,43,44,45]. Besides, recombination events can occur among OPVs since it has been observed in VACV co-cultures and between VARV and CPXV [46,47,48]. Together, these studies suggest that poxviruses from the same species exist as distinct genetical groups and can recombine inside co-infected hosts, which increases the importance of studying the genetic variability among strains and clones of poxviruses circulating in the environment. Even though studies using vaccine strains clones show that VACV can recombine, there is an absence of data concerning VACV clones from field samples. This work aims to contribute to the understanding about the biological and genetic variability among field VACV clones by using genetic and biological analyses to study the diversity of VACV populations isolated during BV outbreaks.

## 2. Materials and Methods

### 2.1. Ethics Approval

The study was approved by the Committee of Ethics in Animal Use from the Universidade Federal de Minas Gerais (CEUA/UFMG). The animals were anesthetized by intraperitoneal injection of ketamine (Vetnil, São Paulo, Brazil) and xylazine (União Química, São Paulo, Brazil) before the infection and those ones that had lost more than 25% of their initial body weight were euthanized with an overdose of anesthetics.

### 2.2. Cells and Viruses

African green monkey kidney BSC-40 and VERO cells (American type cell culture—ATCC) were maintained in 5% CO_2_ atmosphere at 37 °C in Eagle’s Minimum Essential Medium (MEM) (Gibco BRL, Invitrogen, Carlsbad, CA, USA) supplemented with 5% fetal bovine serum (FBS) (Cultilab, Brazil), 25 µg/mL fungizone (Amphotericin B) (Cristália, São Paulo, Brazil), 500 U/mL penicillin (Cristália, São Paulo, São Paulo, Brazil) and 50 µg/mL gentamicin (Schering-Plough, São Paulo, Brazil). VERO cells were used for viral isolation from clinical specimens and growth. The BSC-40 cells were used for viral clones purification, plaque phenotype, comet phenotype and growth curve assays. Only typical poxviruses cytopathic effects were visualized after inoculation of clinical samples in both cell systems. The VACV Western Reserve (VACV-WR) was gently provided by Dr C. Jungwirth (Universität Würzburg, Würzburg, Germany) and was used as a virulent control in mice assays.

### 2.3. Clinical Samples

**Figure 1:**
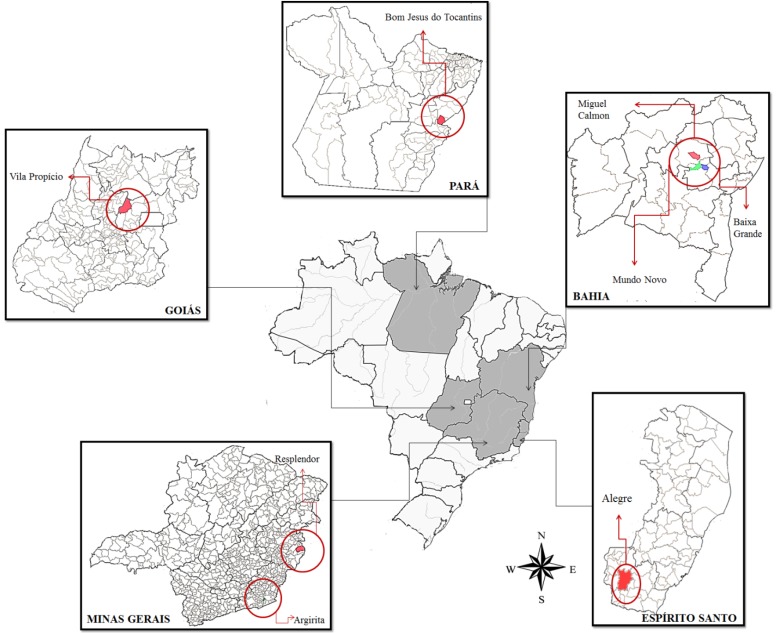
Map displaying Brazilian states and counties from which the clinical samples used for *Vaccinia virus* clone isolation were collected. The red circles indicate the counties where the samples were collected: Miguel Calmon, Baixa Grande and Mundo Novo counties localized in Bahia state, Vila Propício county localized in Goiás state, Bom Jesus do Tocantins county localized in Pará state, Resplendor and Argirita counties localized in Minas Gerais States and Alegre county in Espírito Santo state.

Clinical samples, consisting of swabs of vesicular fluids and scabs obtained from cattle’s teats and milkers’ hands, were collected during BV outbreaks that occurred between 2005 and 2011 in five Brazilian states: Minas Gerais, Bahia, Goiás, Espírito Santo and Pará. Sample collection procedures were performed by veterinarians, following institutional recommendations. Two viruses were isolated from human samples, obtained in Bahia and Minas Gerais states; and eight viruses were isolated from bovine samples, two obtained from Bahia state, two from Para state, two from Goiás state, one from Minas Gerais state and one from Espírito Santo state. The counties and states where the samples were isolated are shown in Table 1 and Figure 1.

**Table 1:** Identification of viral clones isolated from clinical samples.

VACV-BR isolates (abbreviation)	Isolated clones	Source	Isolation state	Isolation year
VACV-BABV	VACV-BABV	*Bos taurus*	Bahia	2011
	Clone 1 to Clone 5			
VACV-BAB2V	VACV-BAB2V	*Bos taurus*	Bahia	2011
	Clone 1 to Clone 5			
VACV-BAHV	VACV- BAHV	*Homo sapiens*	Bahia	2011
	Clone 1 to Clone 5			
VACV-GOBV	VACV-GOBV	*Bos taurus*	Goiás	2011
	Clone 1 to Clone 5			
VACV-GOB2V	VACV-GOB2V	*Bos taurus*	Goiás	2011
	Clone 1 to Clone 5			
VACV-ESBV	VACV-ESBV	*Bos taurus*	Espírito Santo	2008
	Clone 1 to Clone 5			
VACV-MGBV	VACV-MGBV	*Bos taurus*	Minas Gerais	2005
	Clone 1 to Clone 4			
VACV-MGHV	VACV-MGHV	*Homo sapiens*	Minas Gerais	2011
	Clone 1 to Clone 5			
VACV-PABV	VACV-PABV	*Bos taurus*	Pará	2011
	Clone 1 to Clone 5			
VACV-PAB2V	VACV-PAB2V	*Bos taurus*	Pará	2010
	Clone 1 to Clone 4			

### 2.4. Sample Processing and Virus Isolation

Scabs were macerated using a homogenizer (Polytron, Lucerne, Switzerland) in phosphate buffer saline (PBS) (0.1 g scab/0.9 mL PBS) containing 200 U/mL penicillin, 4 µg/mL amphotericin B and 100 µg/mL gentamicin, and then clarified by centrifugation at 2000× *g* for 3 min (min). To the swabs, 200 µL of PBS was added, followed by a centrifugation at 2000× *g* for 3 min [32]. For virus isolation, 200 µL of supernatant was added to VERO cells monolayers which were incubated after adsorption at 37 °C for 72 h or until detection of cytopathic effect (CPE).

### 2.5. Viral Clone Plaque Purification

BSC-40 cells were used for viral plaque purification as described elsewhere [49]. After isolation, each virus isolate was serially diluted (log10) and inoculated into BSC-40 cells monolayers cultured in 6-well plates for clone selection and viral plaque purification. After 1 h of incubation (at 37 °C, 5%CO_2_), culture medium was removed, cell monolayers were washed twice with PBS and solid medium (MEM supplemented with 1% FBS + agarose 1%—Gibco, São Paulo, Brazil) was added. After 48 hours, five viral plaques from each sample were collected and purified in BSC40 cells with solid medium. The criteria used to choose the clones was the plaque size: after measurement, small and large viral plaques were collected from each sample. Individual clones were then isolated by serial plaque-purifications in three passages in BSC40 cells using 1% agarose overlay. The resulting clones were propagated in Vero cells monolayers, purified and titrated as described [49,50]. Isolated VACV strains and clones are shown in Table 1. Two clones, from two distinct clinical samples, were not able to grow after some steps of clone plaque purification (Table 1). After clone plaque purification, clones were manipulated separately to avoid cross-contamination.

### 2.6. Biological Assays

#### 2.6.1. Plaque Phenotype

For plaque phenotype assays, BSC40 cells seeded in 6-well plates at 90%–95% confluence were infected with specific clones. After 1 h of adsorption (37 °C, 5%CO_2_), monolayers were washed twice with PBS and overlaid with solid medium prepared by mixing equal proportion of 1% agarose and 2× Eagle’s minimum essential medium (MEM) (Gibco, São Paulo, Brazil) supplemented with 2% FBS (Gibco, São Paulo, Brazil). After 48 h of incubation (37 °C, 5% CO_2_), cells were fixed with formaldehyde and stained with crystal violet for plaque size analysis.

#### 2.6.2. Comet Phenotype Assay

For comet phenotype assays, BSC40 cells seeded in 6-well plates at 90%–95% confluence were infected with the large plaque clones. After 1 h of adsorption (37 °C, 5% CO_2_), the medium was removed, monolayers were washed twice with PBS and fresh MEM supplemented with 1% FBS was added. After 48 h of incubation (37 °C, 5% CO_2_), the cells were fixed with paraformaldehyde and stained with crystal violet.

#### 2.6.3. Growth Curve Assays

BSC-40 cells were grown in 24-well plates and infected with VACV clones in biological duplicates. Infection was carried out at a MOI (multiplicity of infection) of 0.01 for 0, 12, 24 and 48 h post infection (hpi). Infected monolayers were then harvested, frozen (−70 °C) and thawed (37 °C) three times and titrated in BSC-40 cells as described. The analysis was performed with GraphPad Prism software (Version 5.00, GraphPad Software, San Diego, CA, USA, 2012).

### 2.7. Virulence in BALB/c Mice

To compare the virulence profile of these new clonal VACV isolates, five clones were selected for virulence assays. Mice were housed in filter-top microisolator cages and provided with commercial mouse food and water *ad libitum*. All the animal experiments were carried out in accordance with regulations and guidelines of the Committee of Ethics in Animal Use of the Universidade Federal de Minas Gerais/Brazil. BALB/c mice were anesthetized by intraperitoneal injection of ketamine and xylazine (3.2 mg and 0.16 mg/mice in 0.9% PBS, respectively) before the procedure (dose: 80–120 mg/kg per animal). Groups of four-week old male BALB/c mice (*n* = 4) were inoculated intranasally with 10 µL of viral suspensions containing 10^6^ plaque forming units (pfu) VACV-WR was used as positive control, given its virulence for mice, and the negative control group was inoculated with 10 µL of PBS as described in [25]. Mice were weighed daily, and clinical signs were recorded for 10 days post infection (dpi) [25]. Mice that lost more than 25% of their initial weight were euthanized.

### 2.8. Molecular Assays

#### Amplification of A56R, A26L and C23L Genes and Phylogenetic Analyses

For the clones’ molecular characterization, DNA extractions were carried out using phenol-chloroform-isoamyl alcohol (PCI) [51] and used as template for Polymerase chain reaction (PCR) amplification of the viral genes A56R (hemagglutinin), C23L (chemokine binding protein) and A26L (A-type inclusion body). These genes are traditionally used for OPV phylogenetic studies, including Brazilian VACV strains [27,29,30].

Two different reactions were made for C23L: reaction 1 was made using the primer pair 5'GCGTGTCCCCAGGACAAGGT3' 5' ATGTCGCTGTCTTTCTCTTCTTCGC 3', amplifying a 124 base pairs (bp) DNA fragment found in both VACV-BR groups. Reaction 2 was made with the primer pair 5'GCGTGTCCCCAGGACAAGGT3' and 5'CTGGATGGGTCTTG3', amplifying a 138 bp DNA fragment of VACV-BR Group 2 viruses but not from VACV-BR Group 1 viruses. This is possible since the reverse primer anneals to a region present on group 2 but deleted in group 1. PCR conditions were 94 °C for 10 min, 30 cycles of 94 °C for 30 s, 50 °C for 30 s and 72 °C for 30 s, followed by 72 °C for 10 min. A semi-nested PCR was made for A26L, using the primer pair 5'ACCACGTCTACACTCGGCGA3' and 5'TGCATCGAGAGCGGAGGAGGA3' for the first reaction and the primer pair 5'ACCACGTCTACACTCGGCGA3' and 5'CGATGCCAAGTACATCGACGA3' for the second reaction. Amplicon sizes are 750 bp and 160 bp respectively, and reaction conditions were: 95 °C for 10 min, 30 cycles of 95 °C for 1 min, 60 °C for 1 min and 72 °C for 1 min, followed by 72 °C for 10 min. Reactions were carried out using 10–50 ng of DNA as template with 0.4 mM of each primer; 0.1 M tris-HCl (pH 8.4); 0.5M KCl; 2.5 mM MgCl_2_; 10 mM of each dNTPs and 2U Taq DNA polymerase to a final volume of 20 μL. Brazilian isolates GP1V and GP2V were used as positive controls. Reactions for the A56R gene were made as previously described [52]. PCR products were electrophoresed in 8%-PAGE and silver stained [51].

For sequencing, the A56R gene was amplified with primers and conditions described elsewhere [53], resulting in amplicons of approximately 900 bp. The A56R fragments were sequenced in both orientations and in duplicate in an automated DNA sequencer (ABI PRISM, Applied Biosystems, São Paulo, Brazil). The sequences were aligned with previously published OPV sequences from GenBank (accession numbers can be found in the figures) using MEGA software version 6.1 (Arizona State University, Phoenix, AZ, USA, 2014). The phylogenetic tree based on the A56R gene sequences was constructed by the Maximum Likelihood method based on the Tamura-Nei model implemented in MEGA6.1 (Arizona State University, Phoenix, AZ, USA, 2014). A56R sequences from clonal VACV-BR obtained in this study were deposited in GenBank. (BAB2V-Clone 1: KP282618; BAB2V-Clone 2: KP282619; BAB2V-Clone 3: KP282620; BAB2V Clone 4: KP282621; BAB2V-Clone 5: KP282622; MGHV-Clone 1: KP282623; MGHV-Clone 2: KP282624; MGHV-Clone 3: KP282625; MGHV-Clone 4: KP282626; MGHV-Clone 5: KP282627; BAHV-Clone 1: KP282628; BAHV-Clone 2: KP282629; BAHV-Clone 3: KP282630; BAHV-Clone 4: KP282631; BAHV-Clone 5: KP282632; ESBV-Clone 1: KP282633; ESBV-Clone 2: KP282634; ESBV-Clone 3: KP282635; ESBV-Clone 4: KP282636; ESBV-Clone 5: KP282637; GOB2V-Clone 1: KP282638; GOB2V-Clone 2: KP282639; GOB2V-Clone 3: KP282640; GOB2V-Clone 4: KP282641; GOB2V-Clone 5: KP282642; MGBV-Clone 1: KP282643; MGBV-Clone 2: KP282644; MGBV-Clone 3: KP282645; MGBV-Clone 4: KP282646; PAB2V-Clone 1: KP282647; PAB2V-Clone 2: KP282648; PAB2V-Clone 3: KP282649; PAB2V-Clone 4: KP282650; BABV-Clone 1: KP282651; BABV-Clone 2: KP282652; BABV-Clone 3: KP282653; BABV-Clone 4: KP282654; BABV-Clone 5: KP282655; GOBV-Clone 1: KP282656; GOBV-Clone 2: KP282657; GOBV-Clone 3: KP282658; GOBV-Clone 4: KP282659; GOBV-Clone 5: KP282660; PABV-Clone 1: KP282661; PABV-Clone 2: KP282662; PABV-Clone 3:KP282663; PABV-Clone 4: KP282664; PABV-Clone 5: KP282665).

## 3. Results

### 3.1. Viral Clone Isolation and Biological Assays

**Figure 2:**
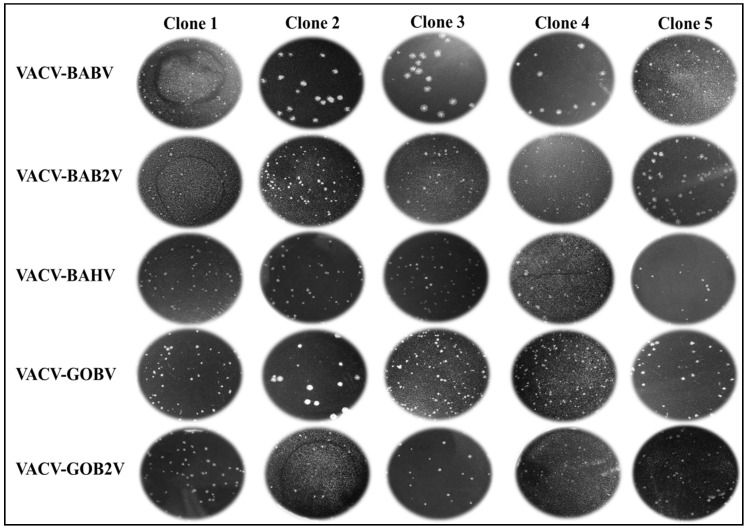
Viral clone plaque phenotype assays. BSC40 cells were cultured in a 6-well dish and then infected with VACV clones. Infection was carried out in the presence of 0.5% agarose for 48 h, followed by fixation and staining. All clones of the VACV-BAB2V, VACV-BAHV and VACV-GOB2V presented only small plaques, while VACV-BABV and VACV-GOBV strains presented small plaque clones as well as large plaque clones. VACV-BABV presented two small plaque clones (Clone 1 and 5) and three large plaque clones (Clone 2, 3 and 4). VACV-GOBV presented four small plaque clones (Clone 1, 3, 4 and 5) and one large plaque clone (Clone 2).

Upon inoculation onto Vero cells, the 10 clinical samples presented typical OPV CPEs, such as lytic plaques, cell agglomerates and formation of vacuoles were observed. No changes were observed in negative control cells. Then, a total of 48 viral clones from the 10 clinical samples were selected. It is important to emphasize that a higher prevalence of small plaques was observed during plaque selection. After three rounds of plaque purification, the selected clones were expanded and purified in sucrose cushion. Biological assays such as plaque phenotype, comet phenotype and growth curve assays were performed to compare the isolated clones. Plaque phenotype assays showed that the clones produced distinct plaques size (Figure 2 and Supplementary Figure S1). The VACV-BABV and VACV-GOBV revealed interesting results. Three clones isolated from the VACV-BABV (BABV-Clone 2, BABV-Clone 3 and BABV-Clone 4) showed a large-plaque profile , while two other clones (BABV-Clone 1 and BAB-Clone 5) presented a small-plaque profile; and the VACV-GOBV showed a single clone (GOBV-Clone 2) with a large-plaque profile and four clones (GOBV-Clone 1, GOBV-Clone 3, GOBV-Clone 4 and GOBV-Clone 5) with the small-plaque profile. These results demonstrate the co-circulation of VACV with at least two distinct plaque-size phenotypes in the same VACV population. The clones obtained from the other isolations showed only small plaque phenotype.

**Figure 3:**
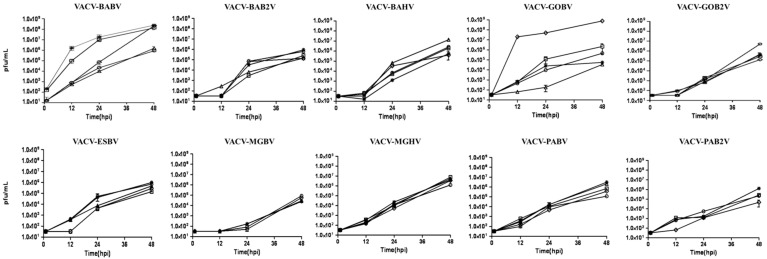
Growth curves assay of VACV-BR clones in BSC-40 cells. Cells were infected at an MOI of 0.01. Viral replication was determined by measuring the viral titer at 0, 12, 24 and 48 h after viral adsorption. The legend is the same for all the graphics: clone 1 (O), clone 2 (◊), clone 3 (*), clone 4 (□), and clone 5 (∆) when present. Each data point represents the average of two independent experiments. The analysis was performed using GraphPad Prism 6.0 software (Version 5.00, GraphPad Software, San Diego, CA, USA, 2012).

To proceed with the biological characterization, the comet phenotype assays for clones were carried out. Prominent comets were observed only in the four large plaque clones (data not shown). In order to compare the growth of VACV clones, we performed a growth curve assay showing that small-plaque clones presented a similar replication profile, but distinct from that observed for large plaque clones, that exhibited an increase of 2–4 logs when compared to the small plaque clones (Figure 3). Thereby, plaque phenotype and growth curve assays showed that large plaque clones display higher replication capacity when compared to small plaque clones and that those different profiles can be found in clones of a same sample.

### 3.2. Virulence of VACV-Clones in Balb/c Mice

**Figure 4:**
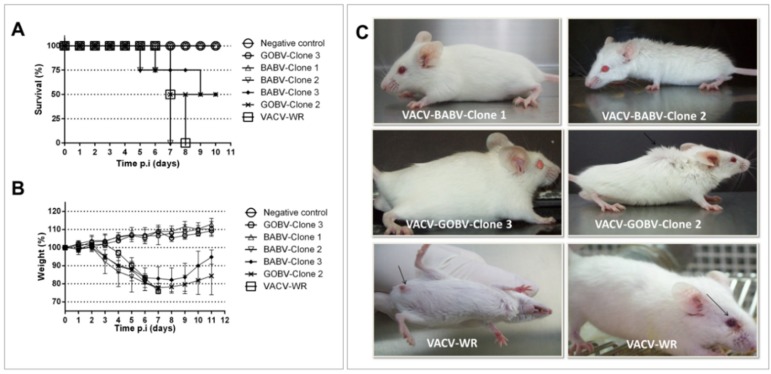
Virulence assays in a BALB/c mice model. Groups of four-week-old mice (*n* = 4) were inoculated by intranasal route with 106 PFU/10 µL of VACV-BABV strain clones 2 and 3 (large-plaque) and clone 1 (small plaque), and VACV-GOBV clone 2 (large plaque) and clone 3 (small plaque). VACV-WR was used as virulent control, and the negative control group was inoculated with PBS. No mice infected with small plaque clones died while the mice infected with large plaque clones had survival rates of 0% and 50%, showing variations in survival rates between the clones (**A**); Uninfected mice (negative control) and animals infected with small plaque clones gained weight, while mice infected with large plaque clones lost weight similarly to mice infected with virulent control VACV-WR (**B**). Ruffling fur and arching back were observed in mice infected with large plaque clones on day 4 p.i. No clinical signals were observed in mice infected with small plaque clones from the same strain. Balanopostitis and periocular alopecy were observed only in mice infected with virulent control VACV-WR on day 3 p.i. No clinical signals were observed in control mice inoculated with PBS.

For virulence assays, we selected the large plaque clones 2 and 3, and the small plaque clone 1 from VACV-BABV, and the large plaque clone 2 and small plaque clone 3 from VACV-GOBV. This selection was based on previous studies that correlated VACV plaque size and virulence in Balb/c mice [25]. Four-week-old BALB/c mice (*n* = 4) were intranasally infected with 1 × 10^6^ pfu of each VACV clone and observed during 10 days for weight change, survival rate and clinical signs. The VACV-WR and PBS were used as virulence-positive and negative controls, respectively. We observed clinical signs, such as ruffling fur and arching back in mice inoculated with large plaque clones and VACV-WR within 3–7 dpi, similar to what has been previously reported for the virulent G2 VACV-BR [25]. However, some clinical signs, such as balanopostitis and periocular alopecia, were only observed in animals infected with VACV-WR (Figure 4C). Survival rates of mice inoculated with large plaque clones ranged from 0% (BABV-clone 2) to 50% (VACV-BABV-clone 3 and VACV-GOBV-clone 2), showing a variation in survival rate among virulent clones (Figure 4A). All mice inoculated with large plaque clones lost weight, while mice inoculated with small-plaque clones and with PBS gained weight over the 10-day experiment (Figure 4B). Moreover, mice inoculated with small-plaque clones, as well as the negative control group, did not present clinical signs and stayed alive during the 10 days of observation similar to what has been previously reported for the non-virulent VACV-BR group 1 [25].

### 3.3. Phylogeny

**Figure 5:**
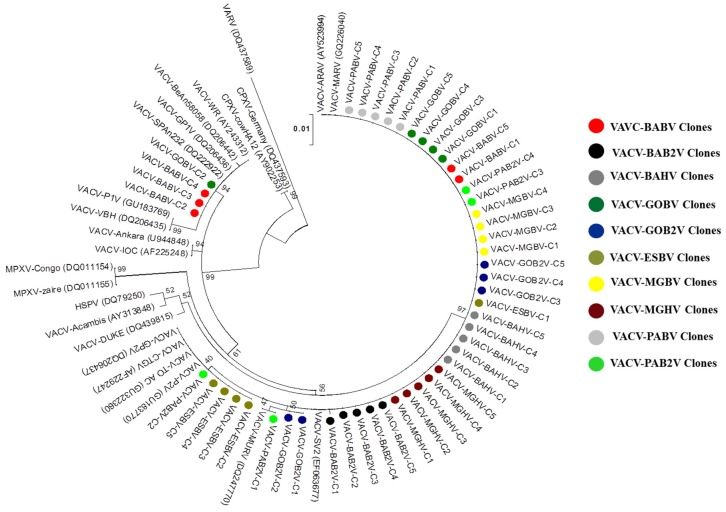
Phylogenetic analysis of viral clones based on the nucleotide sequences of *Orthopoxvirus* A56R gene. The tree was constructed by the Maximum Likelihood method with 1000 bootstrap replicates based in Tamura-Nei model implemented in MEGA6.1 software program (Arizona State University, Phoenix, AZ, USA, 2014) (www.megasoftware.net). Bootstrap confidence intervals are shown on branches. Nucleotide sequences were obtained from GenBank. Distinct colors indicate a strain and its clones as shown in the legend. Clones isolated from the same clinical sample were grouped in separate branches (Groups 1 and 2). Most of the clones were grouped in G1 and subgroups within the G1 were observed.

**Figure 6:**
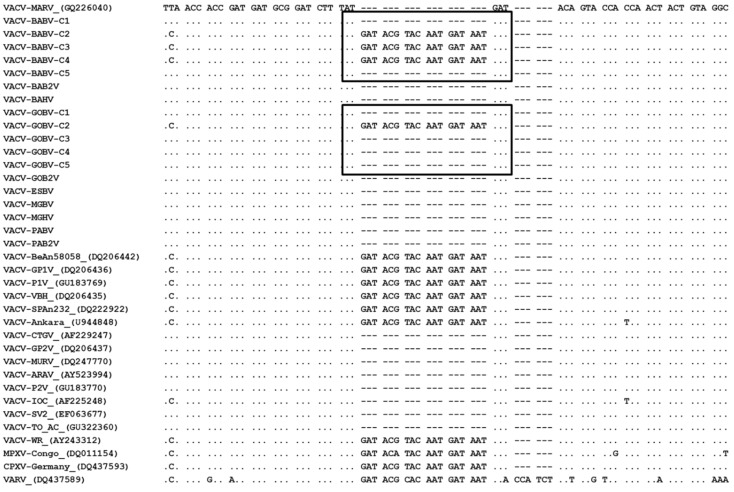
Alignment of a fragment of viral clones A56R gene nucleotide sequences with other *Orthopoxvirus* sequences. The sequences were obtained from GenBank and aligned using the standard parameters of CLUSTAL W. Nucleotide positions are shown according to the VACV-MARV (G1 VACV-BR isolate). (.) indicates identity and (-) indicates deletion of nucleotides. The box highlights significant variations among clones from the same sample in which the clones 1 and 5 from the VACV-BABV showed a deletion of the 18 nucleotides observed in the A56R gene sequence of G1 VACV-BR, while clones 2, 3 and 4 from the same clinical sample did not show this deletion (G2). This alignment displays only representative clones from each sample, except for VACV-BABV and VACV-GOBV, in which all clones are represented.

For molecular comparison of the clones, the A56R, C23L, and A26L genes were amplified. Large plaque clones presented a typical G2 VACV-BR profile while the small plaque clones presented a profile similar to G1, which was corroborated by our biological data. However, two exceptions were found: large plaque clone 2 from VACV-BABV showed a G2 VACV-BR profile for the A56R and C23L genes and G1 profile for the A26L gene, and the small plaque clone 2 from the VACV-MGHV strain showed a G1 VACV-BR profile for the A56R and A26L genes and G2 profile to C23L gene. Table 2 summarizes these results. For phylogeny, we sequenced a partial fragment (900 bp) of the A56R gene of all VACV clones and the obtained sequences were compared to other VACV sequences available at NCBI nucleotide database. Phylogenetic analyses clustered the large plaque clones into G2 VACV-BR branch, while the small plaque clones were branched with G1 VACV-BR, further confirming the biological data (Figure 5). The branches were statistically supported by high bootstrap values, mainly due the presence of a molecular signature of 18-bp deletion in A56R gene from G1 of VACV-BR (Figure 6). Moreover, small plaque clones sequences showed specific polymorphisms that reflected the separation of some clones into different branches within this group (Figure 5 and Supplementary Figure S2).

**Table 2:** VACV-BR group 1 or 2 profile of the isolated clones based on A56R, A26L and C23L genes. Bold font indicates clones with mixed profile (Group 1 and 2).

Clones	Genes			Clones	Genes		
	A56R	A26L	C23L		A56R	A26L	C23L
**BABV-Clone 1**	Group 1	Group 1	Group 1	**GOB2V-Clone 1**	Group 1	Group 1	Group 1
**BABV-Clone 2**	**Group 2**	**Group 1**	**Group 2**	**GOB2V-Clone 2**	Group 1	Group 1	Group 1
**BABV-Clone 3**	Group 2	Group 2	Group 2	**GOB2V-Clone 3**	Group 1	Group 1	Group 1
**BABV-Clone 4**	Group 2	Group 2	Group 2	**GOB2V-Clone 4**	Group 1	Group 1	Group 1
**BABV-Clone 5**	Group 1	Group 1	Group 1	**GOB2V-Clone 5**	Group 1	Group 1	Group 1
**BAB2V-Clone 1**	Group 1	Group 1	Group 1	**ESBV-Clone 1**	Group 1	Group 1	Group 1
**BAB2V-Clone 2**	Group 1	Group 1	Group 1	**ESBV-Clone 2**	Group 1	Group 1	Group 1
**BAB2V-Clone 3**	Group 1	Group 1	Group 1	**ESBV-Clone 3**	Group 1	Group 1	Group 1
**BAB2V-Clone 4**	Group 1	Group 1	Group 1	**ESBV-Clone 4**	Group 1	Group 1	Group 1
**BAB2V-Clone 5**	Group 1	Group 1	Group 1	**ESBV-Clone 5**	Group 1	Group 1	Group 1
**BAHV-Clone 1**	Group 1	Group 1	Group 1	**MGHV-Clone 1**	Group 1	Group 1	Group 1
**BAHV-Clone 2**	Group 1	Group 1	Group 1	**MGHV-Clone 2**	**Group 1**	**Group 1**	**Group 2**
**BAHV-Clone 3**	Group 1	Group 1	Group 1	**MGHV-Clone 3**	Group 1	Group 1	Group 1
**BAHV-Clone 4**	Group 1	Group 1	Group 1	**MGHV-Clone 4**	Group 1	Group 1	Group 1
**BAHV-Clone 5**	Group 1	Group 1	Group 1	**MGHV-Clone 5**	Group 1	Group 1	Group 1
**GOBV-Clone 1**	Group 1	Group 1	Group 1	**PABV-Clone 1**	Group 1	Group 1	Group 1
**GOBV-Clone 2**	Group 2	Group 2	Group 2	**PABV-Clone 2**	Group 1	Group 1	Group 1
**GOBV-Clone 3**	Group 1	Group 1	Group 1	**PABV-Clone 3**	Group 1	Group 1	Group 1
**GOBV-Clone 4**	Group 1	Group 1	Group 1	**PABV-Clone 4**	Group 1	Group 1	Group 1
**GOBV-Clone 5**	Group 1	Group 1	Group 1	**PABV-Clone 5**	Group 1	Group 1	Group 1
**MGBV-Clone 1**	Group 1	Group 1	Group 1	**PAB2V-Clone 1**	Group 1	Group 1	Group 1
**MGBV-Clone 2**	Group 1	Group 1	Group 1	**PAB2V-Clone 2**	Group 1	Group 1	Group 1
**MGBV-Clone 3**	Group 1	Group 1	Group 1	**PAB2V-Clone 3**	Group 1	Group 1	Group 1
**MGBV-Clone 4**	Group 1	Group 1	Group 1	**PAB2V-Clone 4**	Group 1	Group 1	Group 1

## 4. Discussion

Bovine vaccinia outbreaks have been increasingly reported in Brazil since 1999 causing economic, social, veterinary and public health problems [11]. Despite the current and historical importance of VACV, little information is available about its circulation, prevalence, origins, maintenance in environment and natural reservoirs.

In this study, we were able to demonstrate the genetic and biologic variability among VACV and even among clones isolated from the same sample. Previous studies have demonstrated the genetic variability present in vaccine preparations of VACV, but in this study we observed the same profile among viruses isolated during BV outbreaks. Our study reinforced the circulation of the two groups (G1 and 2) of VACV-BR, as previously reported [24,26,27,28]. In addition, our data demonstrate the co-circulation of mixed VACV populations in the same sample, as well as a great genetic diversity among VACV-BR G1 clones in the same sample. In addition, we isolated two clones (VACV-BABV-clone 2 and VACV-MGHV clone 2) that presented a mosaic profile, with both G1 and G2 features based on the molecular analysis of A56R, A26L and C23L genes. These clones may have arisen as product of a recombination event.

Previous studies have shown a high recombination rate during a poxvirus co-infection [46,47,48]. Co-circulation of the genetically different clones in the same sample as shown here may favor the recombination generating new genetic variants which can also have influence in virus evolution and pathogenesis. Moreover, genetic changes can favor adaptation to new hosts, such as described for other viruses of the same genus contributing to expand its circulation and host range [5].

Despite the existence of two VACV groups circulating in Brazil, generally G1 viruses has been more frequently isolated when compared to G2 viruses, including this study in which 92% of the isolated clones were grouped in VACV-BR-G1 while only 8% were grouped in VACV-BR-G2 were based on A56R gene. However, we believe that in addition to the higher prevalence of G1 VACV-BR there is a laboratory filter that favors the isolation and characterization of G1 VACV-BR, since the cellular systems commonly used in isolation and characterization, like embryonated chicken eggs and Vero cells, are unable to highlight differences between G1 and 2 such as plaque size variations. Probably, in a mixed sample (which has G1 and 2 viruses), the prevalence of G2 may be lower as was demonstrated in the case of VACV-GOBV sample, which would not allow the identification of the G2 viruses if the sample is directly processed and characterized by conducting PCR from VACV without previous plaque purification in an appropriate cellular system for the identification of both groups. Thus, we suggest that the viruses that have been isolated represent a small part of the diversity of VACV present in the natural environment. Our findings raise more discussions about the origin of VACV in Brazil, reinforcing the notion that VACV-BR viruses may have more than one origin [54].

We confirmed the virulence differences between large plaque and small plaque viruses as demonstrated previously [25,28]. While small plaque viral clones did not cause weight loss or death during infection in BALB/c mice, large plaque clones caused weight loss, clinical signs and death of infected BALB/C mice, showing that virulent and no virulent clones may be present in the same sample.

Here, we also showed that the replication rates varies among clones of the same sample with large plaque clones showing a higher replication rates when compared to small plaque clones isolated from the same sample in BSC40 cells. The importance of the higher replication rates for VACV evolution and maintenance in the environment needs further studies.

Taken together, data regarding G2 clones show exclusive biological and molecular features (including genes profiles, plaque phenotype and mouse clinical signs) distinct to other known large plaque reference samples, including VACV-Western Reserve.

In summary, our study demonstrated the genetic and biological variability between clones isolated from natural circulating VACV-BR in BV outbreaks, evidencing that VACV can contain viral clone subpopulations. These results raise new questions about the diversity and origin of VACV circulating in Brazilian natural and rural environments. Nevertheless, the real implications of different VACV clones’ co-circulation are unknown but we can emphasize the significance in the evolution and maintenance of these viruses in the environment.

## Supplementary Files

Supplementary File 1

Supplementary File 2
