# Supplementary material for: From Lesions to Viral Clones: Biological and Molecular Diversity amongst Autochthonous Brazilian Vaccinia Virus
Source: Viruses. 2015 Mar 16;7(3):1218–37. doi: 10.3390/v7031218 (PMC4379567; doi:10.3390/v7031218)
Supplement: Supplementary File 2 [file viruses-07-01218-s002.pdf]

Supplementary Information

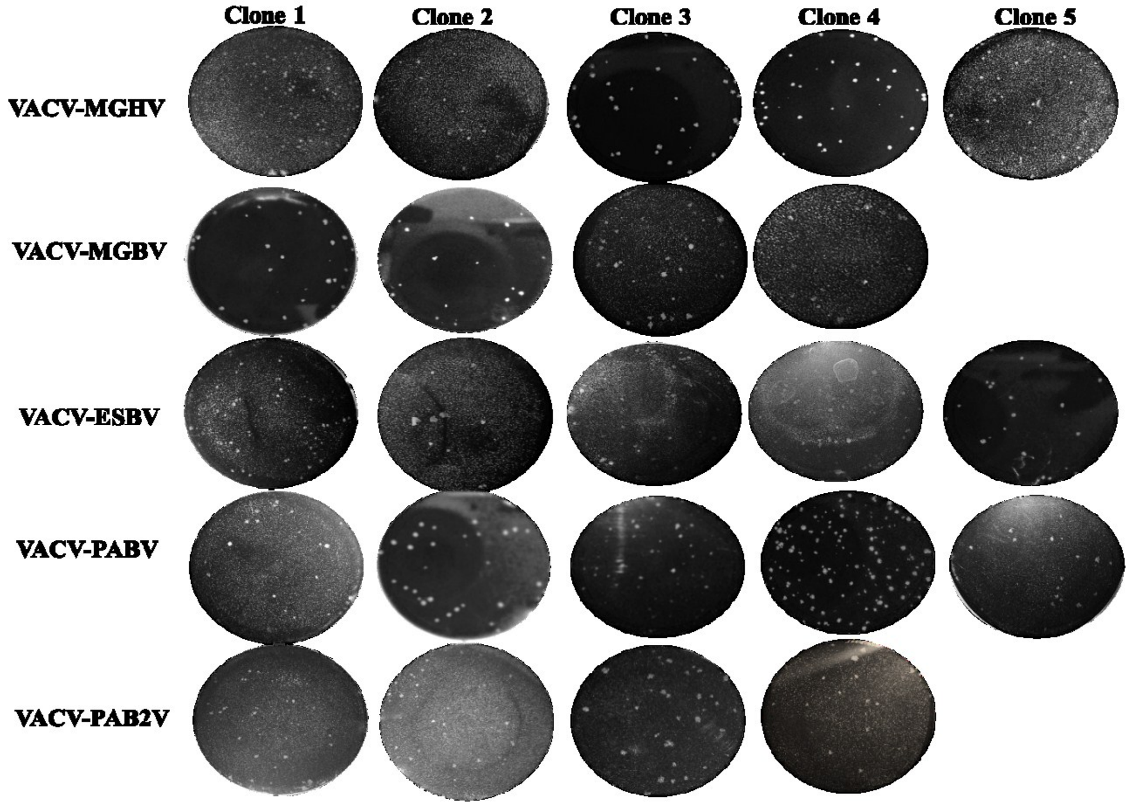

Figure S1. Viral clone plaque phenotype assays. In this figure are displayed the remainder 23 clones (Figure 2 continuation). BSC40 cells were cultured in a 6-well dish and then infected with VACV clones. Infection was carried out in the presence of 0.5% agarose for 48 h, followed by fixation and staining. All clones presented small plaques.

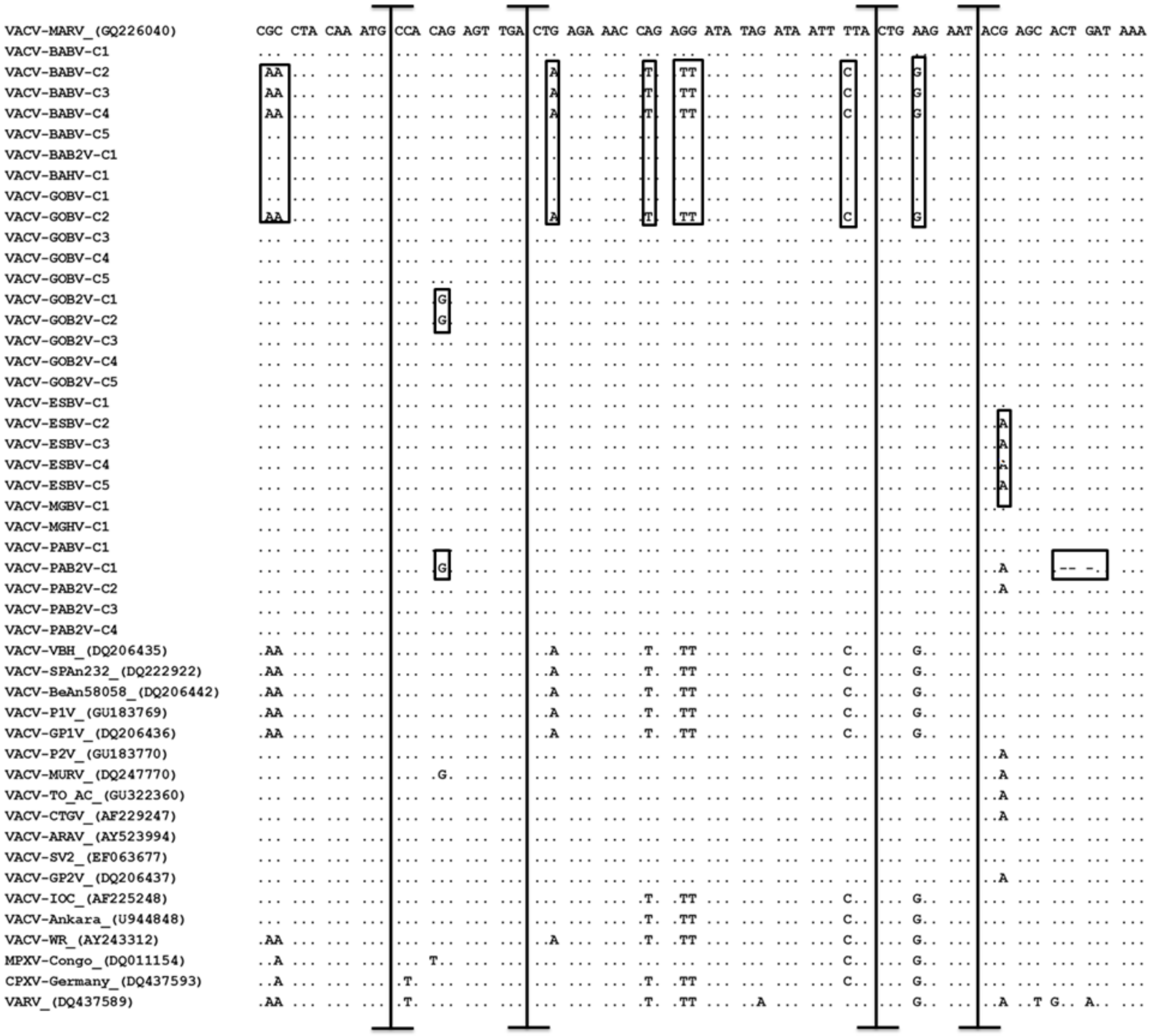

Figure S2. Alignment of different variable regions of A56R nucleotide sequence regions. The sequences were obtained from GenBank and aligned using the default parameters of CLUSTAL W. The nucleotide positions are shown according to the VACV-MARV (G1 member). (.) indicates identity and (-) indicates deletions of nucleotides. The boxes highlight nt substitutions, in which some clones showed 1–8 nt substitutions, and INDELs amongst studied clones.
